# Supplementary material for: Astrocyte redox imbalance underlies prelimbic neuronal hypoactivity and maladaptive affective behaviors in epilepsy
Source: Sci Adv. 2026 Jul 29;12(31):eaef6777. doi: 10.1126/sciadv.aef6777 (PMC13418531; doi:10.1126/sciadv.aef6777)
Supplement: Supplementary file 1 — Figs. S1 to S12 Legend for table S1 [file sciadv.aef6777_sm.pdf]

Science Advances
AAAS

Supplementary Materials for

Astrocyte redox imbalance underlies prelimbic neuronal hypoactivity and maladaptive affective behaviors in epilepsy

Travis E. Faust *et al.*

Corresponding author: Akira Sawa, asawa1@jhmi.edu

Sci. Adv. **12**, eaef6777 (2026)
DOI: 10.1126/sciadv.aef6777

The PDF file includes:

Figs. S1 to S12
Legend for table S1

Other Supplementary Material for this manuscript includes the following:

Table S1

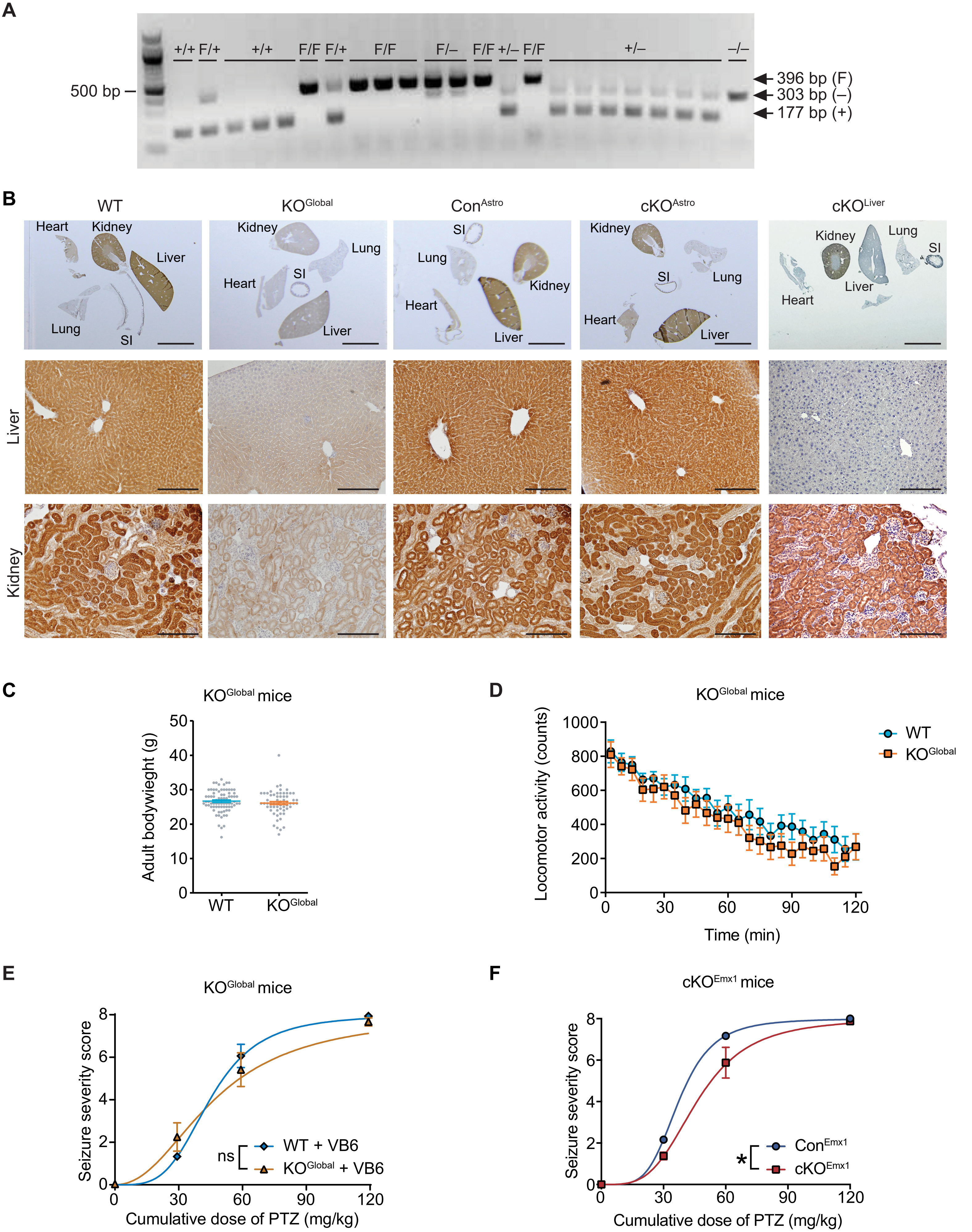

A
+/+
F/+
+/+
F/F
F/+
F/F
F/–
F/F
+/–
F/F
+/–
–/–
500 bp
396 bp (F)
303 bp (–)
177 bp (+)
B
WT
KO^Global
Con^Astro
cKO^Astro
cKO^Liver
Heart
Kidney
Liver
Lung
SI
Liver
Kidney
C
KO^Global mice
Adult bodywieght (g)
WT
KO^Global
D
KO^Global mice
Locomotor activity (counts)
Time (min)
WT
KO^Global
E
KO^Global mice
Seizure severity score
Cumulative dose of PTZ (mg/kg)
ns
WT + VB6
KO^Global + VB6
F
cKO^Emx1 mice
Seizure severity score
Cumulative dose of PTZ (mg/kg)
*
Con^Emx1
cKO^Emx1

Fig. S1. Molecular validation and baseline behavior of ALDH7A1 knockout mice.

(**A**) Gel electrophoresis of PCR products from *Aldh7a1* floxed mice. Bands corresponding to floxed (F), wild type (+), and knockout (–) alleles are indicated.
(**B**) Representative ALDH7A1 DAB staining in peripheral tissues (heart, kidney, liver, lung, and small intestine (SI)) from WT, KO^{Global}, Con^{Astro}, cKO^{Astro}, and cKO^{Liver} mice. Lower panels show high magnification images of liver and kidney. Scale bars 5 mm (top), 200 μm (bottom).
(**C**) Adult bodyweight in KO^{Global} and WT mice (n=62-78 mice; P>0.05)
(**D**) Locomotor activity in KO^{Global} and WT mice during the open field test (n=11-14 mice; genotype effect P>0.05)
(**E**) PTZ seizure threshold test in KO^{Global} and WT mice on pyridoxine (VB6)-supplemented diet. Lines represent fitted dose-response curves (n=12-15 mice; ns:P>0.05).
(**F**) PTZ seizure threshold test in cKO^{Emx1} and Con^{Emx1} mice. Lines represent fitted dose-response curves (n=6-8 mice; *P<0.05).
Data represent mean ± S.E.M.
Statistics: Student's t-test (C); 2-way repeated measures ANOVA (D); Extra sum-of-squares F test on EC_{50} (E, F).

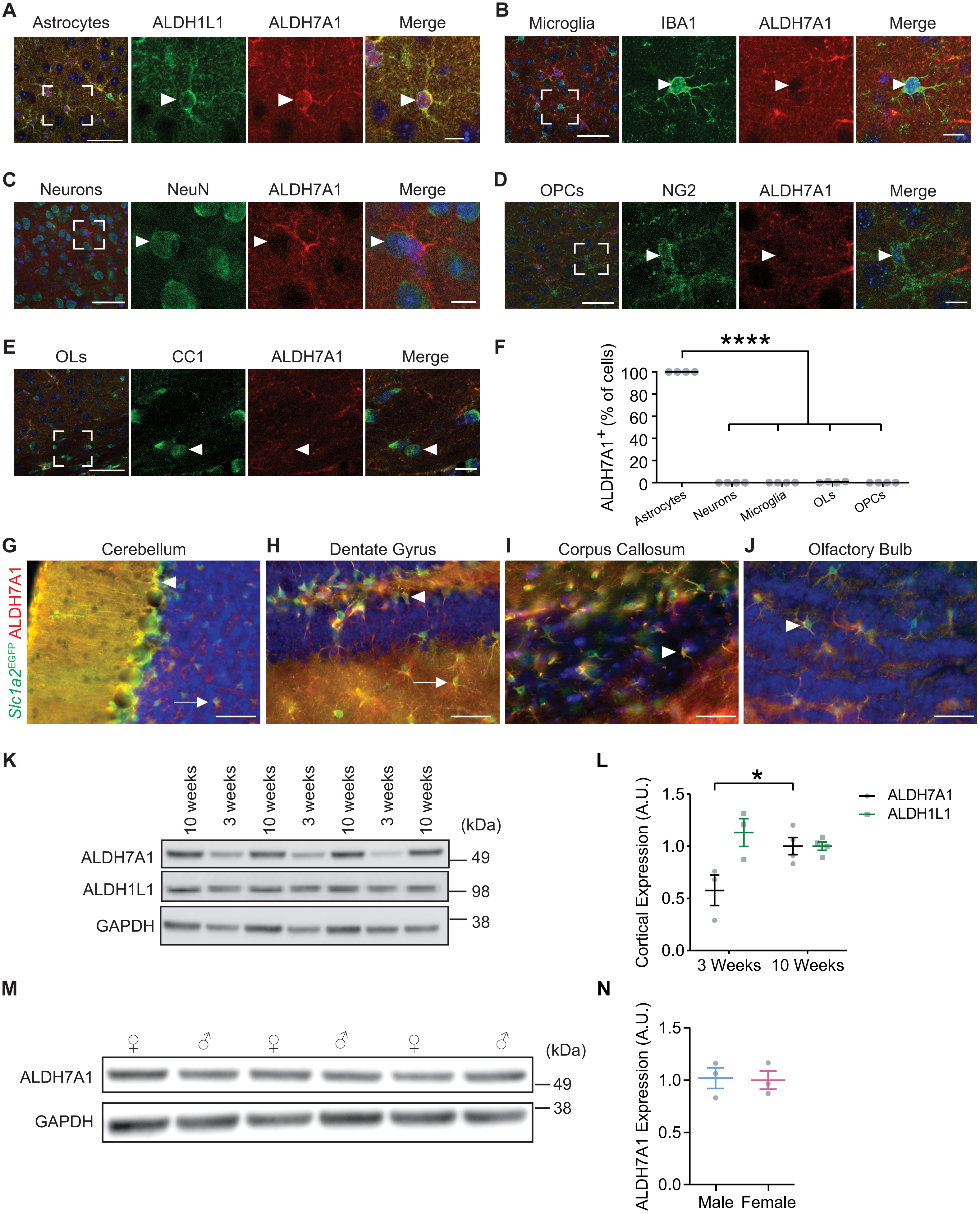

A
Astrocytes
ALDH1L1
ALDH7A1
Merge
B
Microglia
IBA1
ALDH7A1
Merge
C
Neurons
NeuN
ALDH7A1
Merge
D
OPCs
NG2
ALDH7A1
Merge
E
OLs
CC1
ALDH7A1
Merge
F
****
ALDH7A1+ (% of cells)
100
80
60
40
20
0
Astrocytes
Neurons
Microglia
OLs
OPCs
G
Cerebellum
H
Dentate Gyrus
I
Corpus Callosum
J
Olfactory Bulb
Slc1a2EGFP ALDH7A1
K
10 weeks
3 weeks
10 weeks
3 weeks
10 weeks
3 weeks
10 weeks
(kDa)
ALDH7A1
ALDH1L1
GAPDH
49
98
38
L
*
Cortical Expression (A.U.)
1.5
1.0
0.5
0.0
3 Weeks
10 Weeks
ALDH7A1
ALDH1L1
M
♀
♂
♀
♂
♀
♂
(kDa)
ALDH7A1
GAPDH
49
38
N
ALDH7A1 Expression (A.U.)
1.5
1.0
0.5
0.0
Male
Female

Fig. S2. ALDH7A1 expression is not heterogeneous across brain regions or sexes.

(**A**-**E**) Representative ALDH7A1 immunofluorescence in adult WT cortex co-labeled with ALDH1L1 (A), IBA1 (B), NeuN (C), NG2 (D), and CC1 (E). Insets show higher magnification views Arrowheads indicate representative labeled cells. Scale bars 50 µm; insets scale bars 10 µm.
(**F**) Percentage of astrocytes (ALDH1L1), neurons (NeuN), microglia (IBA1), oligodendrocytes (OLs; CC1), and oligodendrocyte precursor cells (OPCs; NG2) expressing ALDH7A1 in adult mouse cortex (*n*=4 mice; ****P<0.0001).
(**G**-**J**) Representative ALDH7A1 immunofluorescence (red) in cerebellum (G), dentate gyrus (H), corpus callosum (I), and olfactory bulb (J) of adult *Slc1a2*$^{EGFP/+}$ mice. Arrows and arrowheads indicate colocalization with distinct GFP^+ astrocyte populations. Scale bars 100 µm.
(**K**-**L**) ALDH7A1 proteins levels in cortical homogenates at 3 and 10 weeks of age. (K) Representative Western blot; (L) quantification of ALDH7A1 and ALDH1L1 normalized to GAPDH (*n*=3-4 mice; *P<0.05).
(**M**-**N**) ALDH7A1 protein levels in adult male and female cortex. (M) Representative Western blot; (N) Quantification normalized to GAPDH (*n*=3 mice; P>0.05).
Data represent mean ± S.E.M.
Statistics: 1-way repeated measure ANOVA with Tukey post-hoc test (F); 2-way repeated measures ANOVA with Holm-Sidak post-hoc test (L); Student's t-test (N).

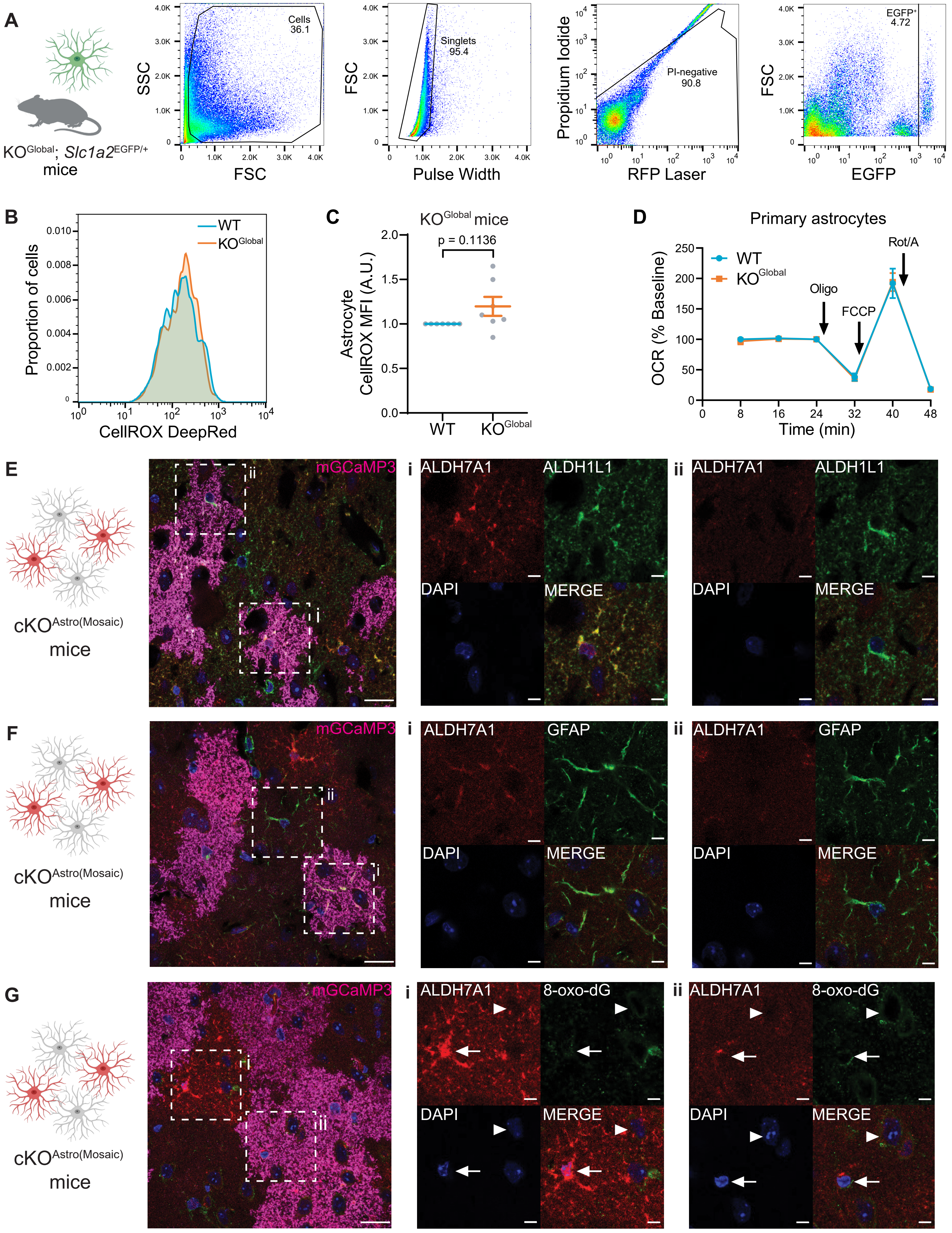
A
B
C
D
E
F
G
KO^Global; Slc1a2^EGFP/+ mice
SSC
FSC
Cells 36.1
Singlets 95.4
Pulse Width
Propidium Iodide
RFP Laser
PI-negative 90.8
EGFP+ 4.72
EGFP
Proportion of cells
CellROX DeepRed
WT
KO^Global
KO^Global mice
p = 0.1136
Astrocyte CellROX MFI (A.U.)
Primary astrocytes
OCR (% Baseline)
Time (min)
Oligo
FCCP
Rot/A
cKO^Astro(Mosaic) mice
mGCaMP3
ALDH7A1
ALDH1L1
GFAP
8-oxo-dG
DAPI
MERGE
i
ii

Fig. S3. ALDH7A1 deletion does not induce astrocyte reactivity or robust oxidative stress.

(**A**) Gating strategy for acutely isolated astrocytes from adult KO^{Global}; *Slc1a2*$^{EGFP/+}$ mice.
(**B**) Representative histograms of CellROX DeepRed fluorescence in astrocytes isolated from WT and KO^{Global} littermates.
(**C**) Fold change in CellROX mean fluorescence intensity in astrocytes isolated from KO^{Global} mice compared to paired WT controls (n=7 mice; P=0.1136).
(**D**) Oxygen consumption rate (OCR) of primary astrocytes from KO^{Global} and WT mice during a Seahorse mitochondrial stress test. Arrows indicate addition of oligomycin (1 μM), FCCP (2 μM), and rotenone + antimycin (0.5 μM each) (n=3 independent cultures; P>0.05).
(**E**-**G**) Experimental schematic (left) and representative immunofluorescence (right) of ALDH7A1 (red) and mGCaMP3 (magenta) in cortex of $cKO^{Astro(Mosaic)}$ mice co-labeled with ALDH1L1 (E), GFAP (F), and 8-oxo-dG (G). Insets show higher magnification images of $ALDH7A1^{+}$ and $ALDH7A1^{-}$ astrocytes. In (G), nuclear 8-oxo-dG signal is indicated in an astrocyte (arrow) and a neighboring cell (arrowhead). Scale bars 20 μM; inset scale bars 5 μM.
Data represent mean ± S.E.M.
Statistics: 1-sample t-test (C); 2-way repeated measures ANOVA with Holm-Sidak post-hoc tests (D).
Graphic elements created in BioRender. Faust, T. (2026) https://BioRender.com/dslslnp.

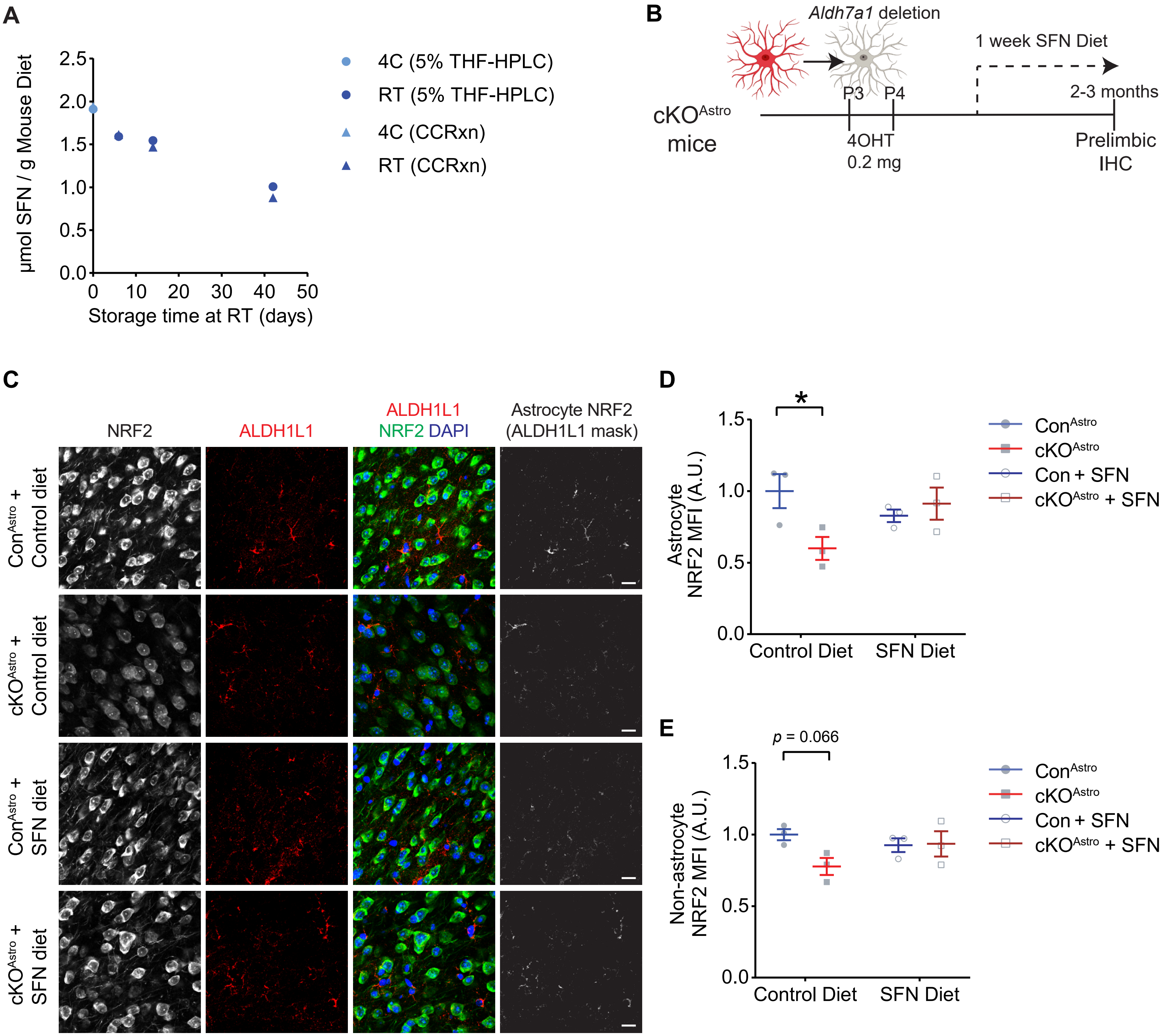
A
µmol SFN / g Mouse Diet
2.5
2.0
1.5
1.0
0.5
0.0
0
10
20
30
40
50
Storage time at RT (days)
4C (5% THF-HPLC)
RT (5% THF-HPLC)
4C (CCRxn)
RT (CCRxn)
B
Aldh7a1 deletion
1 week SFN Diet
cKOAstro mice
P3
P4
2-3 months
4OHT 0.2 mg
Prelimbic IHC
C
NRF2
ALDH1L1
ALDH1L1 NRF2 DAPI
Astrocyte NRF2 (ALDH1L1 mask)
ConAstro + Control diet
cKOAstro + Control diet
ConAstro + SFN diet
cKOAstro + SFN diet
D
*
Astrocyte NRF2 MFI (A.U.)
1.5
1.0
0.5
0.0
Control Diet
SFN Diet
ConAstro
cKOAstro
Con + SFN
cKOAstro + SFN
E
p = 0.066
Non-astrocyte NRF2 MFI (A.U.)
1.5
1.0
0.5
0.0
Control Diet
SFN Diet
ConAstro
cKOAstro
Con + SFN
cKOAstro + SFN

Fig. S4. Validation of sulforaphane diet.

(**A**) Concentration of sulforaphane (SFN) in mouse diet pellets stored long-term at 4 °C or left at room temperature for 6, 14, or 42 days. Individual measurements by direct chromatography (%5 THF-HPLC) and by cyclocondensation (CCRxn) are shown.
(**B**) Experimental schematic for 1-week dietary SFN treatment
(**C**-**E**) Representative NRF2 and ALDH1L1 immunofluorescence in the prelimbic cortex of cKO^{Astro} and Con^{Astro} mice on control diet or after 1 week of SFN treatment (C). Right panels show NRF2 signal within $ALDH1L1^{+}$ astrocytes (ALDH1L1 mask). Scale bars 20 μm. NRF2 mean fluorescent intensity in $ALDH1L1^{+}$ astrocytes (D) and outside $ALDH1L1^{+}$ astrocytes (E) (n=3 mice; *P<0.05, P=0.066).
Data in (D-E) represent mean ± S.E.M.
Statistics: 2-way repeated measures ANOVA with Holm-Sidak post-hoc tests (D, E).
Graphic elements created in BioRender. Faust, T. (2026) https://BioRender.com/1zopr86.

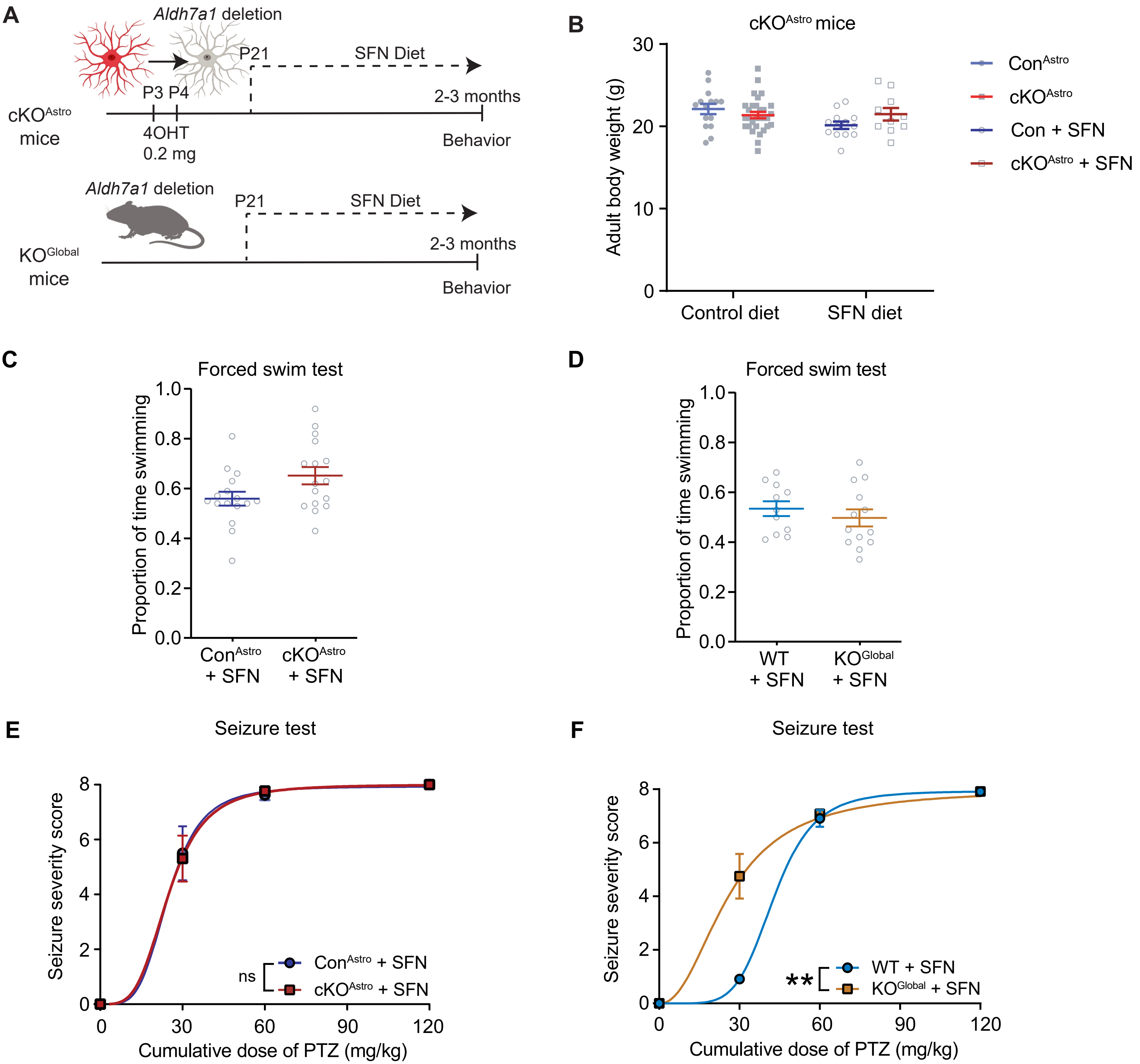
A
Aldh7a1 deletion
P21
SFN Diet
P3 P4
2-3 months
cKO^Astro
mice
4OHT
0.2 mg
Behavior
Aldh7a1 deletion
P21
SFN Diet
2-3 months
KO^Global
mice
Behavior
B
cKO^Astro mice
Adult body weight (g)
30
20
10
0
Control diet
SFN diet
Con^Astro
cKO^Astro
Con + SFN
cKO^Astro + SFN
C
Forced swim test
Proportion of time swimming
1.0
0.8
0.6
0.4
0.2
0.0
Con^Astro + SFN
cKO^Astro + SFN
D
Forced swim test
Proportion of time swimming
1.0
0.8
0.6
0.4
0.2
0.0
WT + SFN
KO^Global + SFN
E
Seizure test
Seizure severity score
8
6
4
2
0
ns
Con^Astro + SFN
cKO^Astro + SFN
0
30
60
90
120
Cumulative dose of PTZ (mg/kg)
F
Seizure test
Seizure severity score
8
6
4
2
0
**
WT + SFN
KO^Global + SFN
0
30
60
90
120
Cumulative dose of PTZ (mg/kg)

Fig. S5. Behavioral analysis of sulforaphane diet.

(**A**) Experimental schematic for long-term dietary sulforaphane (SFN) treatment.
(**B**) Adult bodyweight in cKO^{Astro} and Con^{Astro} mice on control or SFN diet (*n*=10-30 mice; *P*>0.05).
(**C**-**D**) Forced swim test: time swimming in cKO^{Astro} and Con^{Astro} mice (C) and KO^{Global} and WT mice (D) on SFN diet (*n*=11-16 mice; *P*>0.05).
(**E**-**F**) PTZ seizure threshold test in cKO^{Astro} and Con^{Astro} mice (E) and KO^{Global} and WT mice (F) on SFN diet. Lines represent fitted dose-response curves (*n*=8-13 mice; ns:*P*>0.05, ***P*<0.01).
Data represent mean ± S.E.M.
Statistics: 2-way ANOVA with Holm-Sidak post-hoc tests (B); Student's t-test (C, D); Extra sum-of-squares F test on EC_{50} (E, F).
Graphic elements created in BioRender. Faust, T. (2026) https://BioRender.com/2lojd94.

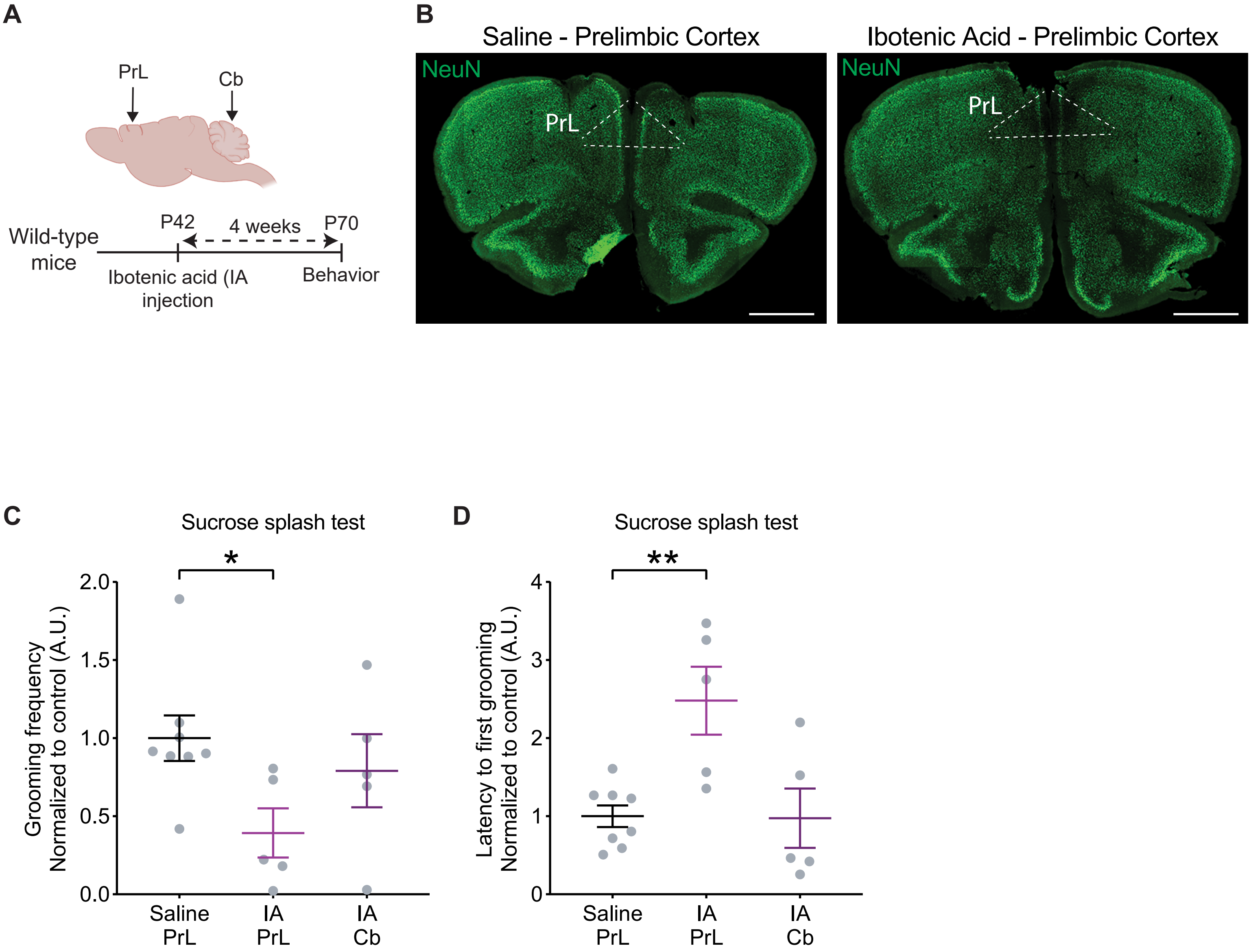
A
PrL
Cb
Wild-type mice
P42
4 weeks
P70
Ibotenic acid (IA injection
Behavior
B
Saline - Prelimbic Cortex
Ibotenic Acid - Prelimbic Cortex
NeuN
PrL
C
Sucrose splash test
Grooming frequency Normalized to control (A.U.)
Saline PrL
IA PrL
IA Cb
D
Sucrose splash test
Latency to first grooming Normalized to control (A.U.)
Saline PrL
IA PrL
IA Cb

Fig. S6. Pharmacological lesioning of prelimbic cortex impairs sucrose splash behavior.

(**A**) Experimental schematic for pharmacological lesioning with ibotenic acid.
(**B**) Representative NeuN immunolabeling in prelimbic cortex following ibotenic acid or saline injection. Scale bars 1 mm.
(**C**-**D**) Sucrose splash: grooming frequency (C) and latency to grooming (D) in WT mice following saline or ibotenic acid (IA) injection into prelimbic cortex (PrL) or cerebellum (Cb) (n=5-8 mice; *P<0.05, **P<0.05).
All data represent mean ± S.E.M.
Statistics: One-way ANOVA with Dunnett's post-hoc test (C, D).
Graphic elements created in BioRender. Faust, T. (2026) https://BioRender.com/hqhf0hm.

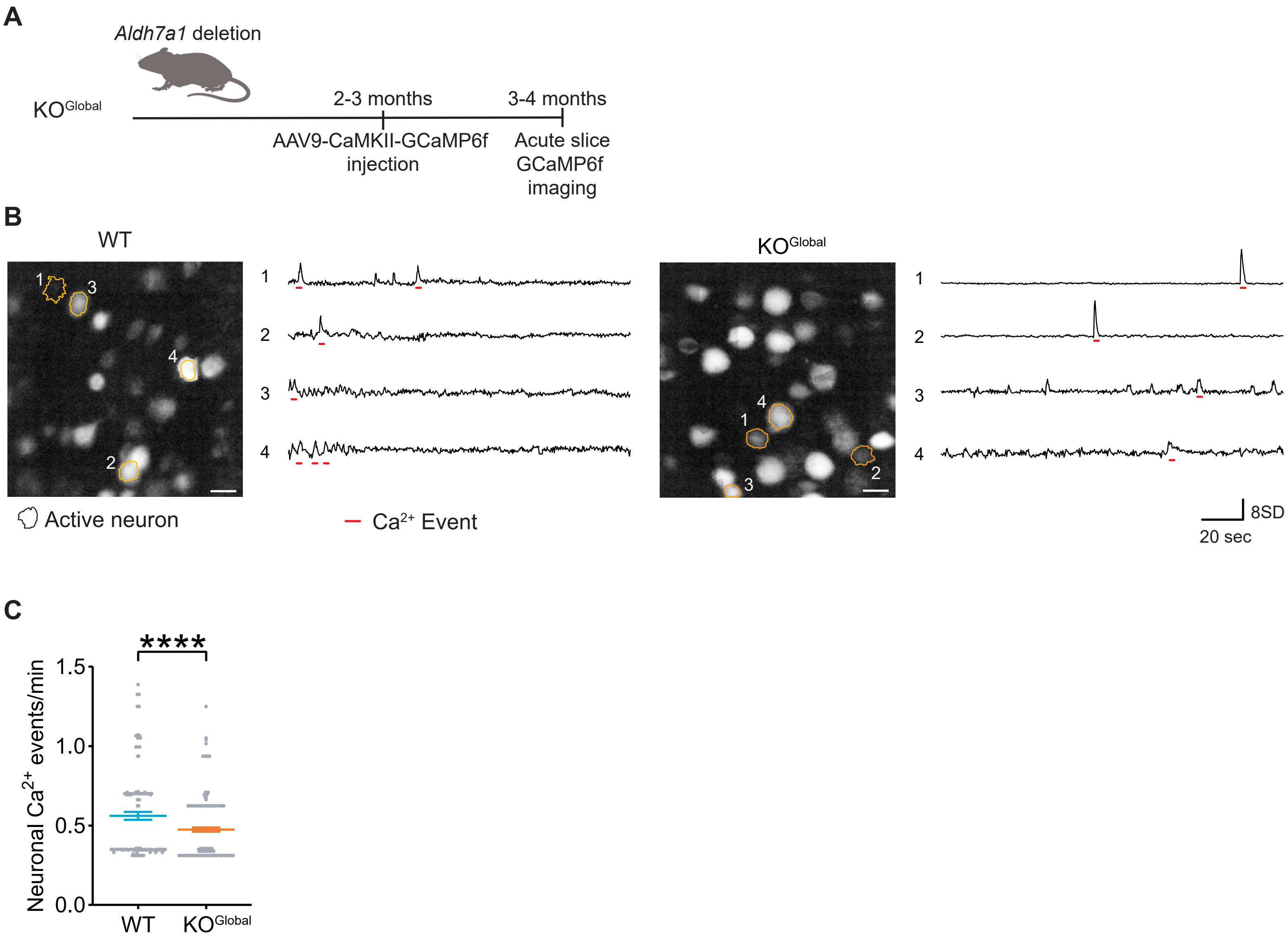
A
Aldh7a1 deletion
KOGlobal
2-3 months
3-4 months
AAV9-CaMKII-GCaMP6f injection
Acute slice GCaMP6f imaging
B
WT
KOGlobal
Active neuron
Ca2+ Event
8SD
20 sec
C
****
Neuronal Ca2+ events/min
1.5
1.0
0.5
0.0
WT
KOGlobal

Fig. S7. Reduced layer 5 pyramidal neuron activity in the prelimbic cortex of KO^{Global} mice.

(**A**) Experimental strategy to assess prelimbic layer 5 (L5) pyramidal neuron activity by GCaMP6f fluorescence in KO^{Global} and WT mice.
(**B**) Representative images (left) and traces (right) of GCaMP6f fluorescence in prelimbic cortex of KO^{Global} and WT mice. Numbered outlines indicate neurons in images used for analysis and shown in traces. Events are indicated by red underlines. Scale bars 20 µm.
(**C**) Prelimbic L5 pyramidal neuron Ca^{2+} event frequency in WT and KO^{Global} mice (n=139-209 neurons; ****P<0.0001).
Data represent mean ± S.E.M.
Statistics: Mann-Whitney test (C).
Graphic elements created in BioRender. Faust, T. (2026) https://BioRender.com/fdyt9wr.

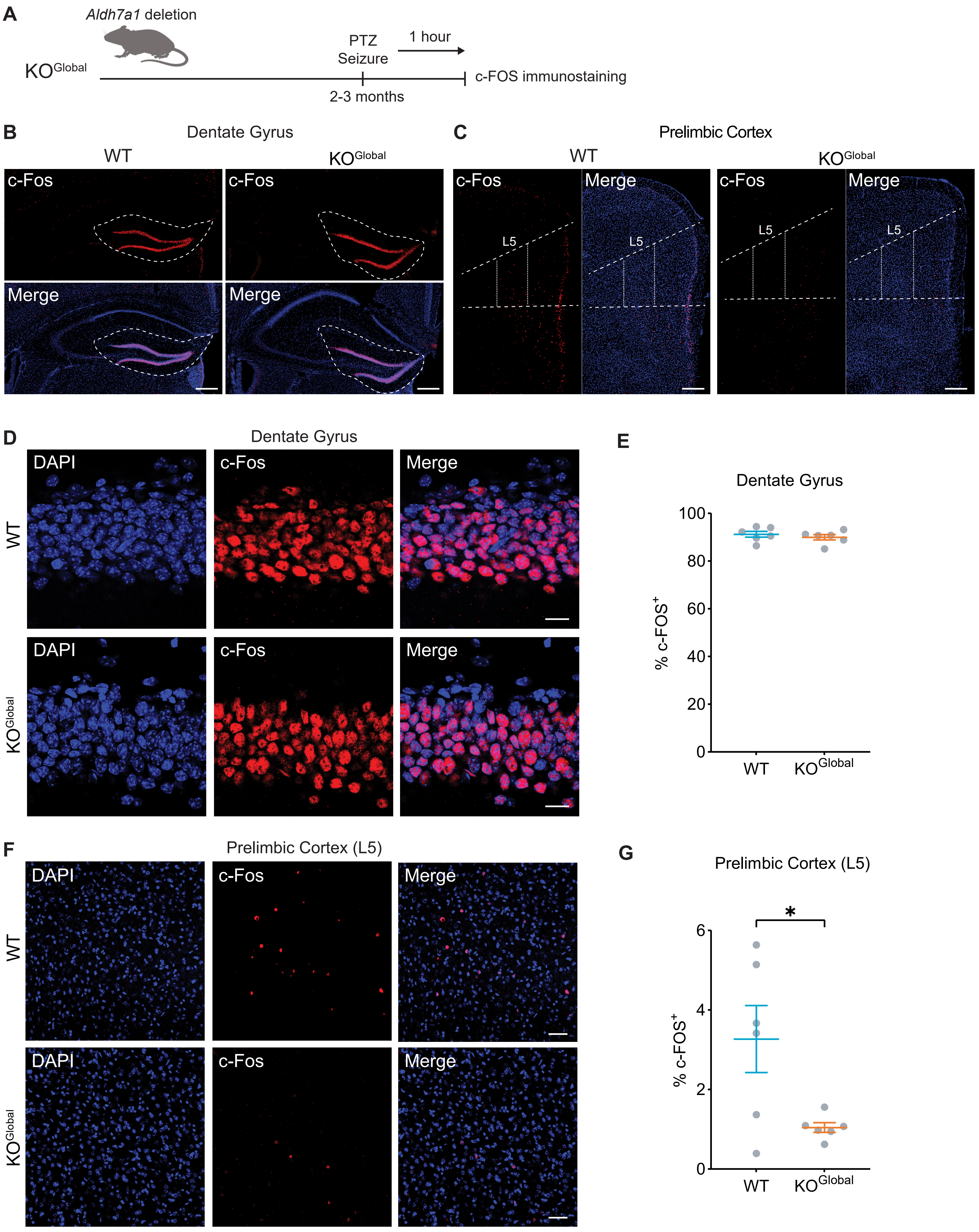
A
Aldh7a1 deletion
KO^Global
PTZ
Seizure
1 hour
c-FOS immunostaining
2-3 months
B
Dentate Gyrus
WT
KO^Global
c-Fos
Merge
C
Prelimbic Cortex
L5
D
DAPI
E
% c-FOS+
100
80
60
40
20
0
F
Prelimbic Cortex (L5)
G
*
6
4
2

Fig. S8. KO^{Global} mice have a reduced percentage of c-FOS^{+} cells in the prelimbic cortex during seizures.

(**A**) Experimental strategy to assess neuronal activity by c-FOS immunofluorescence in KO^{Global} and WT mice 1 hour after PTZ-induced seizures.
(**B**-**C**) Representative c-FOS immunofluorescence in hippocampus (B) and prelimbic cortex (C) of KO^{Global} and WT mice. White dashed outlines indicate dentate gyrus in (B) and layer 5 (L5) prelimbic cortex in (C). Scale bars 200 μm.
(**D**-**E**) Representative immunofluorescence (D) and quantification (E) of c-FOS^{+} cells in dentate gyrus of KO^{Global} and WT mice 1 hour post-seizure (n=6 mice; P>0.05).
(**F**-**G**) Representative immunofluorescence (F) and quantification (G) of c-FOS^{+} cells in L5 prelimbic cortex of KO^{Global} and WT mice 1 hour post-seizure (n=6 mice; *P<0.05).
Data represent mean ± S.E.M.
Statistics: Student's t-test (E, G).
Graphic elements created in BioRender. Faust, T. (2026) https://BioRender.com/i8xog6x.

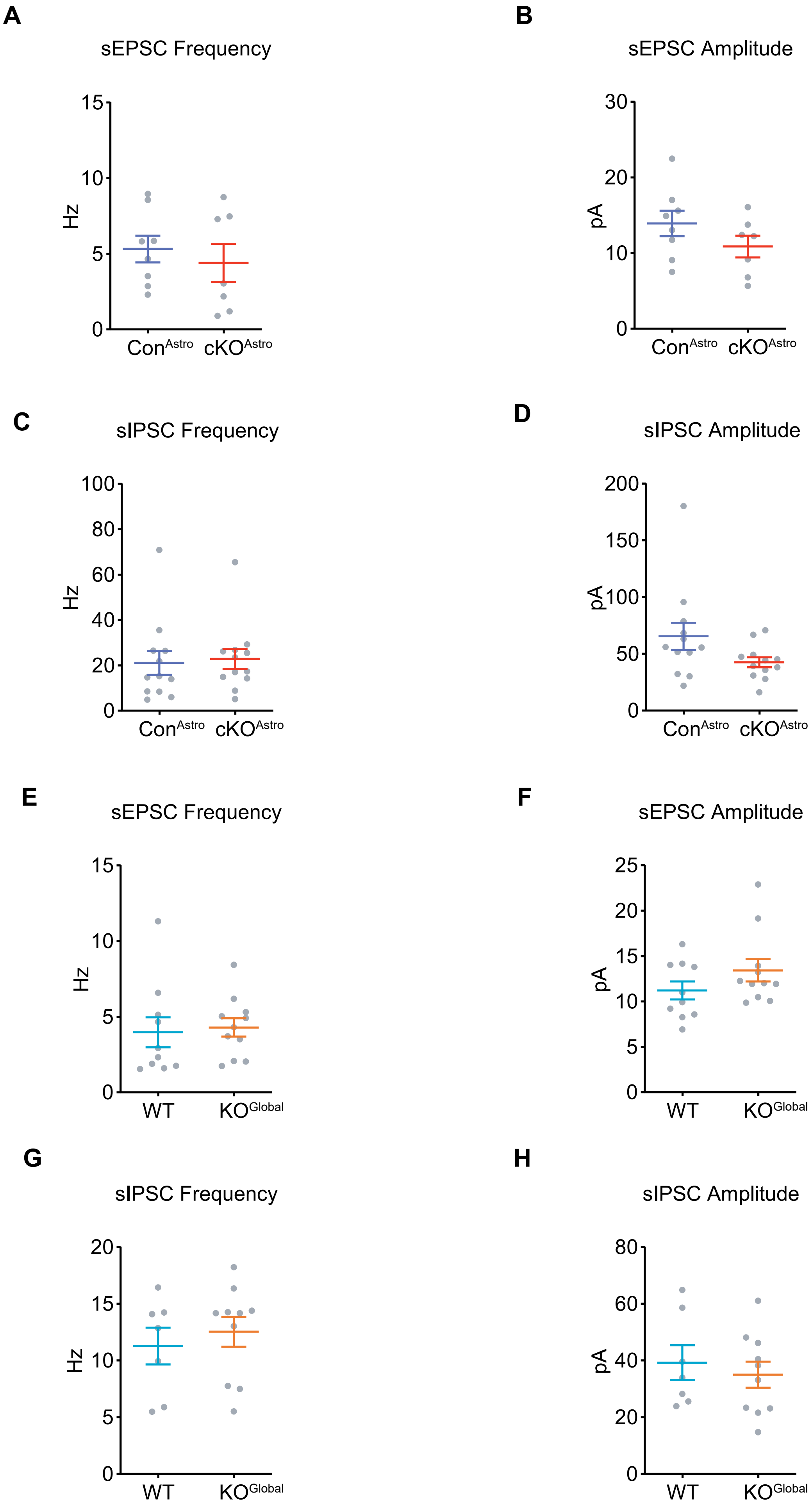
A
sEPSC Frequency
Hz
15
10
5
0
Con^{Astro} cKO^{Astro}
B
sEPSC Amplitude
pA
30
20
10
0
Con^{Astro} cKO^{Astro}
C
sIPSC Frequency
Hz
100
80
60
40
20
0
Con^{Astro} cKO^{Astro}
D
sIPSC Amplitude
pA
200
150
100
50
0
Con^{Astro} cKO^{Astro}
E
sEPSC Frequency
Hz
15
10
5
0
WT KO^{Global}
F
sEPSC Amplitude
pA
25
20
15
10
5
0
WT KO^{Global}
G
sIPSC Frequency
Hz
20
15
10
5
0
WT KO^{Global}
H
sIPSC Amplitude
pA
80
60
40
20
0
WT KO^{Global}

Fig. S9. No changes in spontaneous postsynaptic currents on layer 5 pyramidal neurons in the prelimbic cortex of cKO^{Astro} or KO^{Global} mice.

(**A**-**D**) Frequency and amplitude of spontaneous excitatory postsynaptic currents (sEPSCs; A, B) and spontaneous inhibitory postsynaptic currents (sIPSCs; C, D) in prelimbic layer 5 pyramidal neurons from cKO^{Astro} and Con^{Astro} mice (n=7-12 cells; P>0.05).
(**E**-**H**) Frequency and amplitude of sEPSCs (E, F) and sIPSCs (G, H) in prelimbic layer 5 pyramidal neurons from KO^{Global} and WT mice (n=7-11 cells; P>0.05).
Data represent mean ± S.E.M.
Statistics: Student's t-test (A-H).

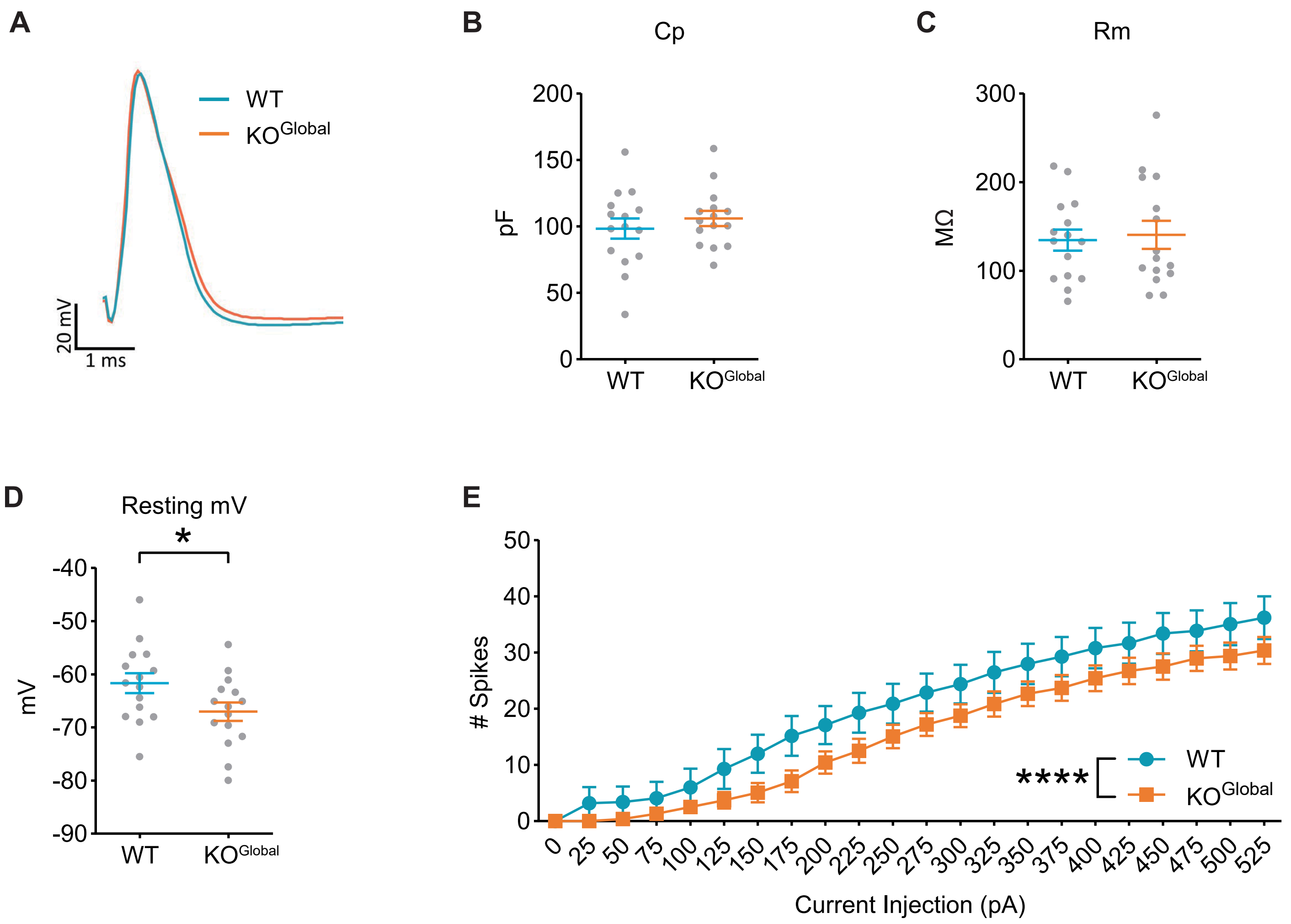

A
WT
KOGlobal
20 mV
1 ms
B
Cp
pF
200
150
100
50
0
WT
KOGlobal
C
Rm
MΩ
300
200
100
0
WT
KOGlobal
D
Resting mV
*
mV
-40
-50
-60
-70
-80
-90
WT
KOGlobal
E
Spikes
50
40
30
20
10
0
****
WT
KOGlobal
0 25 50 75 100 125 150 175 200 225 250 275 300 325 350 375 400 425 450 475 500 525
Current Injection (pA)

Fig. S10. Reduced resting membrane voltage and excitability of layer 5 pyramidal neurons in the prelimbic cortex of KO^{Global} mice.

(**A**) Representative action potential traces from prelimbic layer 5 pyramidal neurons in KO^{Global} and WT mice.
(**B**-**D**) Membrane capacitance (B), membrane resistance (C), and resting membrane voltage (D) of prelimbic layer 5 pyramidal neurons in KO^{Global} and WT mice (n=15 cells; *P<0.05).
(**E**) Number of spikes generated per current injection in prelimbic layer 5 pyramidal neurons in KO^{Global} and WT mice (n=10-15 cells; main effect of genotype: **** P < 0.0001).
Data represent mean ± S.E.M.
Statistics: Student's t-test (B-D); 2-way repeated measures ANOVA with Holm-Sidak post-hoc test (E).

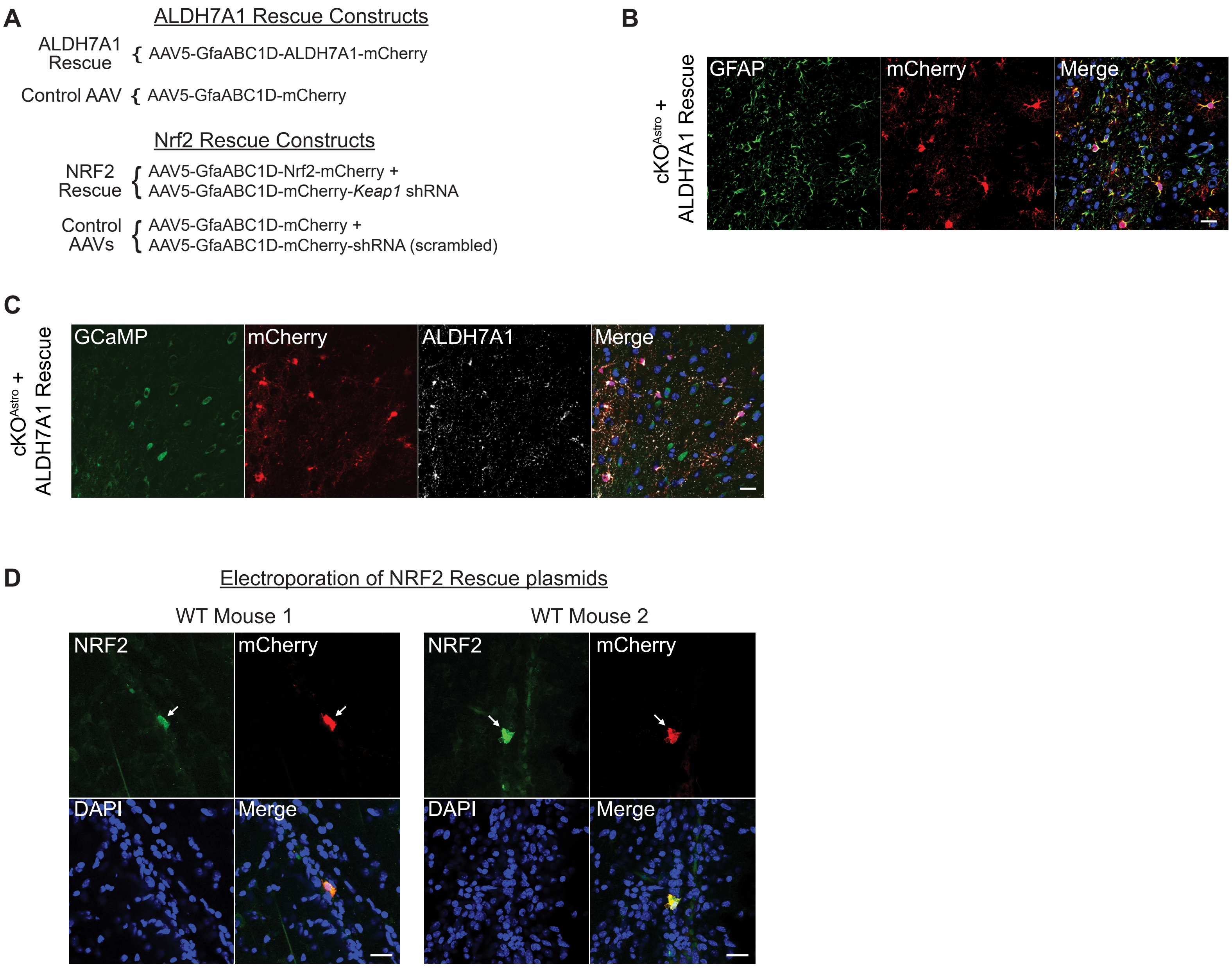

A
ALDH7A1 Rescue Constructs
ALDH7A1 Rescue
AAV5-GfaABC1D-ALDH7A1-mCherry
Control AAV
AAV5-GfaABC1D-mCherry
Nrf2 Rescue Constructs
NRF2 Rescue
AAV5-GfaABC1D-Nrf2-mCherry +
AAV5-GfaABC1D-mCherry-Keap1 shRNA
Control AAVs
AAV5-GfaABC1D-mCherry +
AAV5-GfaABC1D-mCherry-shRNA (scrambled)
B
cKOAstro + ALDH7A1 Rescue
GFAP
mCherry
Merge
C
cKOAstro + ALDH7A1 Rescue
GCaMP
mCherry
ALDH7A1
Merge
D
Electroporation of NRF2 Rescue plasmids
WT Mouse 1
NRF2
mCherry
DAPI
Merge
WT Mouse 2
NRF2
mCherry
DAPI
Merge

Fig. S11. Validation of ALDH7A1 and NRF2 Rescue constructs.

(**A**) Schematic of ALDH7A1 and NRF2 Rescue constructs and respective controls.
(**B**-**C**) Representative immunofluorescent images from the prelimbic cortex of cKO^{Astro} mice injected with ALDH7A1 Rescue AAV (AAV5-GfaABC1D-ALDH7A1-mCherry) showing astrocyte marker GFAP (green) and viral marker mCherry (red) (B), and GCaMP (green), ALDH7A1 (white), and viral marker mCherry (red) (C). Scale bars 20 µm.
(**D**) Representativ NRF2 (green) and mCherry (red) immunofluorescence in WT mice electroporated with pZac2.1-GfaABC1D-Nrf2-mCherry and pZac2.1-GfaABC1D-mCherry-*Keap1* shRNA. Scale bars 20 µm.

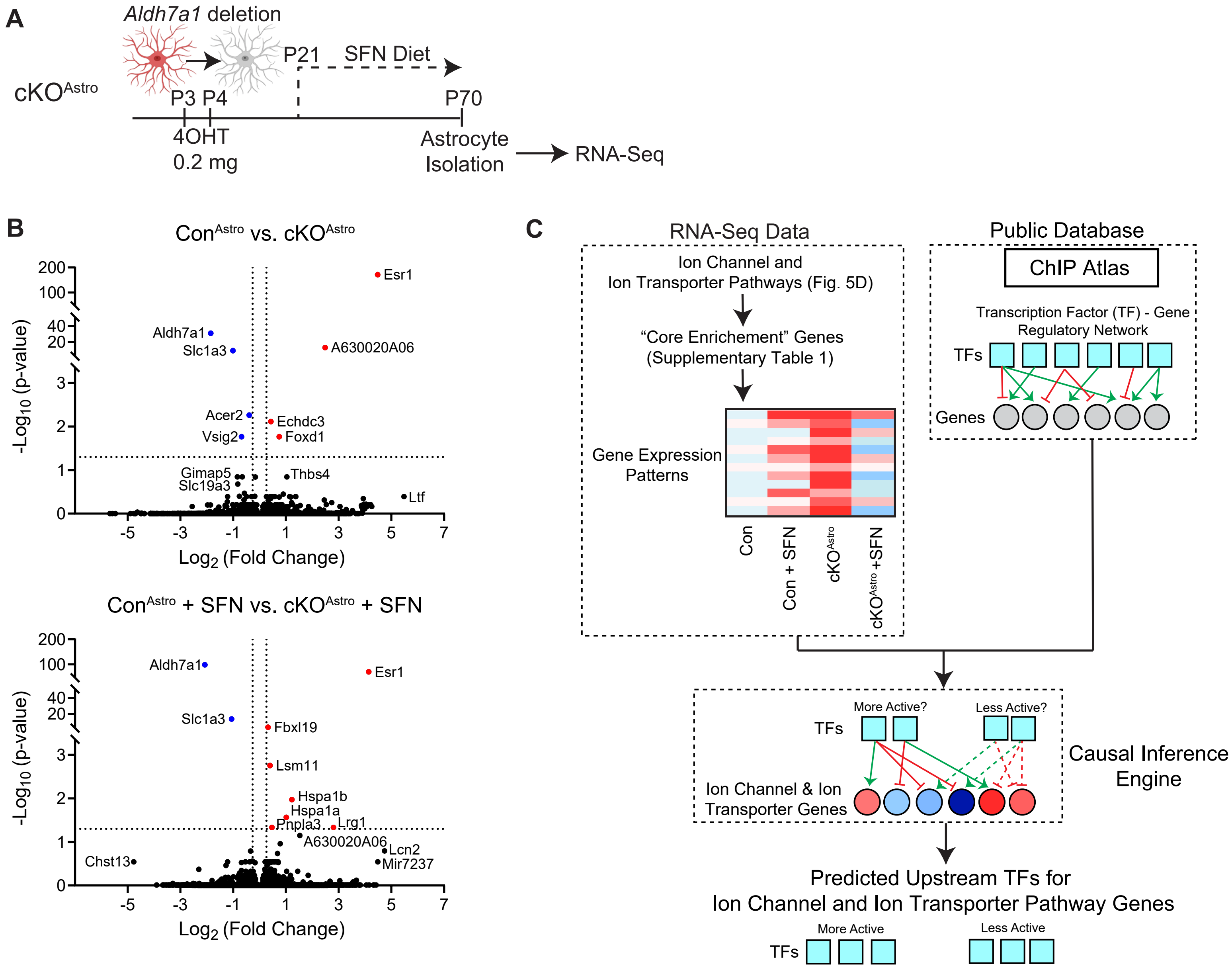

D

Comparison	Predicted by CIE
cKO^{Astro} vs. Con^{Astro}	Yes
cKO^{Astro} + SFN vs. Con^{Astro}	No
Con^{Astro} + SFN vs. Con^{Astro}	No

Predicted Upstream TFs for
Ion Channel and Ion Transporter Pathway Genes

More Active

ZBTB17 ZEB1 TET2 BRD2

GREB1 FOXF2 SMAD1

Less Active

GATA4

Fig. S12. Putative upstream regulators of ion channel and ion transporter gene changes in cKO^{Astro} astrocytes include redox-sensitive transcription factors.

(**A**) Experimental schematic for RNA sequencing of cortical astrocytes isolated from cKO^{Astro}, cKO^{Astro} + SFN, Con^{Astro}, and Con^{Astro} + SFN mice.
(**B**) Volcano plots of RNA sequencing results comparing astrocytes isolated from cKO^{Astro} vs. Con^{Astro} and cKO^{Astro} + SFN vs Con^{Astro} + SFN mice. Dotted lines indicate cutoffs at $P<0.05$ and $\log_2$(fold change)>|1.2|.
(**C**) Schematic summary of causal inference engine (CIE) analysis identifying transcription factors upstream of ion channel and ion transporter genes across conditions.
(**D**) Table summarizing the filtering strategy used to identify transcription factors altered in cKO^{Astro} astrocytes and rescued by SFN, and transcription factors fitting these criteria.
Graphic elements created in BioRender. Faust, T. (2026) https://BioRender.com/e989d1l.

Table S1. Bioinformatic analysis of RNA sequencing results.

Excel file containing the results of RNA sequencing analyses of cortical tissue from KO^{Global} mice (Sheets 2-4, related to Figure 2) and of astrocytes isolated from cKO^{Astro}, Con^{Astro}, cKO^{Astro} + SFN, and Con^{Astro} + SFN mice (Sheets 5-11, related to Figures 5 and S8). Analyses include DESeq2 differential gene expression, GSEA pathway analysis of gene ontology terms, and causal interference engine (CIE) analysis of upstream regulators.
